# Comparison of agrochemicals allocation efficiency between greenhouse and open-field vegetables in China

**DOI:** 10.1038/s41598-021-92316-7

**Published:** 2021-06-17

**Authors:** Yinghui Yuan, Xiaoheng Zhang

**Affiliations:** 1 School of Horticulture and Landscape Architecture, Jinling Institute of Technology, Nanjing, 210038 China; 2 College of Economics and Management, Nanjing University of Aeronautics and Astronautics, Nanjing, 211106 China

**Keywords:** Environmental economics, Sustainability, Environmental impact

## Abstract

The overuse of agrochemicals in greenhouse production system has aroused high concerns in China. Existing studies have investigated the status and determinants of agrochemicals overuse for greenhouse vegetables whereas little is known about whether the agrochemicals are allocated efficiently from economic perspective. We use a translog production function and the inputs and outputs data of vegetable production in 34 Chinese cities during 2004–2017 to calculate agrochemicals allocation efficiency for both open-field and greenhouse vegetables. We find that the agrochemicals are allocated inefficiently due to overuse for both open-field and greenhouse vegetables, whereas the overuse degree of chemical fertilizer used in greenhouse vegetables is lower than that of open-field vegetables during a growing duration. In addition, we also find that the application levels of agrochemicals for greenhouse vegetables per mu (15 mu = 1 hectare) are higher than those of open-field vegetables, but the application levels of agrochemicals for per kilogram greenhouse vegetables are significantly lower. We conclude that the overuse of agrochemicals in greenhouse production system may attribute to the year-round production of greenhouse vegetables induced by economic incentives. Therefore, reducing the number of production rotations may be an effective method to alleviate the overuse of agrochemicals in greenhouse vegetables.

## Introduction

Greenhouse production system plays essential roles in China’s vegetable production. According to the statistics of Food and Agriculture Organization of the United Nations, China produced 171.9 million tons of vegetables in 2017, accounting for 51.2% of the global total production^1^. High production of vegetables in China not only meets the domestic needs of nearly 1.4 billion people, but also can be exported to international market–China exported 15.5 billion dollars of vegetables in 2017^2^. How can China achieve such superior performance in vegetable production? Apart from the abundant labor resources to produce vegetables, the wide application of greenhouse in China is a more important reason. Greenhouse can provide regulated and controlled environment (especially temperature and illumination) to produce vegetables regardless of location and season. Besides the typically multiple cropping rotations within a year, greenhouse system can prolong the harvest time of one cropping rotation, and even produce off-season vegetables, thus harvesting more outputs compared to open-field production system. The sown areas and productions of greenhouse vegetables accounted for 21.5% and 30.5% of China’s total vegetable sown areas and productions, respectively^3^. Furthermore, greenhouse production system produced more than 60% of the output value of Chinese vegetable industry and provided farmers with 70 million jobs^3,4^.

Despite the great contribution to vegetable industry, greenhouse system has aroused many concerns due to its serious environmental problems, especially when compared to open-field system. Greenhouse vegetable production system is known to result in accumulation of heavy metals, trace elements and phthalate esters in soils^5–10^, acidification and salinization of soils^11–13^, large N_2_O emission^14^, severe contamination of groundwater and irrigation water^15,16^, as well as the decrease in soil enzyme activities and microbial functional diversity^17^. In addition, greenhouse production system also increases the potential health risks to both farmers and consumers due to exposing to the pollutants and pollutants residual in vegetables^8,18,19^.

A large number of studies have investigated the status and determinants of agrochemicals overuse of greenhouse vegetables in China^11, 20-24^. More specifically, farmers often used up to about 5 times the greenhouse vegetables requirement in the case of N fertilizer in Beijing^11^. Hanafi^25^ documented that lots of pesticides are used in greenhouse because of the diseases and insect pests infection caused by high temperature and humidity. The study by Yu et al.^12^ found that the special microclimate environment in greenhouse, such as the strong evaporation of soil water and a lack of rainfall leaching, together with the inappropriate farming methods, including flood irrigation and excessive application of agrochemicals, have resulted in low fertilizer use efficiency. However, greenhouse could produce vegetables with less pesticide and fertilizer by adopting modern technology and management practices such as biological control, closed watering system, and carefully directed fertilization^26–28^.

Exploring how farmers allocate the agrochemicals plays core role in deeply understanding the overuse of agrochemicals and the pollution caused by agrochemicals. The quantities of agrochemicals application are determined by vegetable farmers whose targets are to maximize their profit given the technology and input prices. Therefore, we cannot make comparison of agrochemical use efficiency directly between greenhouse and open-field vegetables because greenhouse vegetables usually have higher sale price and yield than open-field vegetables^3,4^.

Following Hopper’s^29^ method, this paper aims at evaluating the allocation efficiency of agrochemicals used in greenhouse and open-field vegetables by calculating the marginal product values (MPVs) of agrochemicals and making comparison with the agrochemicals’ prices. Farmers would not think they overuse the agrochemicals in production process if the value produced by additional unit agrochemical is larger than its corresponding cost. Therefore, compared to open-field vegetables, farmers could apply more agrochemicals to greenhouse vegetables because the decreased marginal value of agrochemicals could be offset by the higher product price. Based on the results of this paper, we would answer whether agrochemicals used in greenhouse vegetables are more but allocative efficient, just like the hypothesis of “poor but efficient” proven by Hopper^29^. The hypothesis of “poor but efficient”proven by Hopper means that although the farmers in India is very poor, but they allocate their input efficiently because the value of an input’s marginal product equals to its price. Therefore, although Chinese greenhouse vegetables used more agrochemicals than open-field vegetables, the allocation efficiency of agrochemicals may be higher than that of open-field vegetables because of higher yield and sale prices of greenhouse vegetables. Given the high concerns of agrochemicals overused by greenhouse vegetables, uncovering the allocative efficiency of agrochemicals would provide significant implications for agricultural policy.

There are at least three methods to measure the use efficiency of fertilizers and pesticides. First, the ratio of the minimum possible input in theory to the observed input is defined as use efficiencies^30–33^, which can be used to evaluate the overuse status of fertilizers or pesticides. Second, another indicator for use efficiency widely accepted by many countries is the fraction of input harvested as product^34^. Zhang et al.^35^ converted the quantities of multiple pesticides with the same functions into an index and compared the index with the recommended range to identify the use efficiency. According to the aforementioned definitions, only the quantities of inputs and outputs are used in these two measurements which do not take the differentiated prices between greenhouse vegetables and open-field vegetables into consideration. The goal of a farmer is to maximize his profits based on market price of vegetables when he makes decisions on fertilizers or pesticides application. Therefore, this paper will calculate the marginal product values and makes the comparisons of the MPVs of chemical fertilizer, organic fertilizer and pesticide with their corresponding prices to identify the allocative efficiency of agrochemicals. The equality of one input’s MPV and its price indicates that farmers allocate this input efficiently, and the sign and magnitude of an inequality mean the direction and severity of the overuse. This method is widely adopted by agricultural economists to study the input’s allocative efficiency^29,36–39^.

To calculate the MPVs of agrochemicals, we have to estimate the production function. To the extent that fertilizer and pesticide have different influential mechanism on vegetable production, thus treating the inputs symmetrically would result in biased estimation of production function^40–42^ and lead to erroneous MPVs. Lichtenberg and Zilberman^40^, Zhang et al.^42^, Paris^43^, Chambers and Lichtenberg^44^, and Sun et al.^45^ have revealed that pesticide plays a damage-abating role in production process whereas other inputs act as the productivity-increasing roles. Therefore, the specification of production function is revised in their studies based on the damage-abating role of pesticides. Guan et al.^41^ furtherly pointed out that different categories of inputs contribute to crop yields through different process. Guan et al.^41^ proposed a more general framework by specifying an asymmetric production function. They categorize all of the inputs in crop production into growth inputs and facilitating inputs by integrating both agronomic principles and agricultural economics concepts. Growth inputs are defined as those directly involved in the biological process of crop growth including land, seed, chemical fertilizers and organic fertilizers. Facilitating inputs are those to improve growth conditions including pesticides, capital and labors.

There are two central contributions of this paper. First, to best of our knowledge, this paper firstly evaluates the allocative efficiency of fertilizers and pesticides for both greenhouse vegetables and open-field vegetables by making comparisons of the input’s MPV with its price in China. MPV accounts not only for the impacts of input on output, but also for the effects of differentiated prices between greenhouse and open-field vegetables on input application level. To the extent that evaluating the allocative efficiency of agrochemicals in both greenhouse and open-field vegetables could provide a new insight to understand the overuse of agrochemicals.

Second, based on the authoritative input–output data, this paper evaluates the allocative efficiency of agrochemicals used in both greenhouse and open-field vegetables during a growth duration to rule out the impacts of the continuous cropping model. Greenhouse production system is initially developed to provide regulated and controlled growing condition to produce crops in areas and seasons usually unsuitable for agricultural production. However, farmers may grow vegetables several times in greenhouse per year to harvest more outputs. The overuse of agrochemicals could be significantly reduced if farmers just take advantage of the function to provide controlled growing conditions without year-round production.

## Method

This paper adopts the method developed by Guan et al.^41^ to calculate MPVs of chemical fertilizer and pesticides. For this method, we should firstly estimate the production function, and then use the estimated parametric of chemical fertilizer and pesticide to calculate MPVs.

Both Cobb–Douglas form and Translog form are widely used as the specific form of the production function in empirical studies^32,37^. This paper uses the translog functional form because this form is very flexible and it’s a second-order approximation of any production technology^10,32^. The translog functional form includes the linear terms, the quadratic terms and the interaction terms of all inputs. It is worth noting that the translog functional form treats all of the inputs symmetrically. To get more precise MPVs of fertilizers and pesticides, this paper adopts Guan et al.’s^41^ method which integrates both agronomic principles and agricultural economics concepts by distinguishing between growth inputs and facilitating inputs in production function. The specification of translog production function used in this paper is as follows:

1 $$ \begin{aligned}   \ln Y_{{it}}  &  = a_{0}  + \alpha _{L} \ln L_{{it}}  + a_{S} \ln S_{{it}}  + a_{F} \ln F_{{it}} {\text{ + }}\alpha _{O} \ln O_{{it}}  + \frac{1}{2}a_{{LL}} \left( {\ln L_{{it}} } \right)^{2}  \\     & \quad  + \,a_{{LS}} \ln L_{{it}} \ln S_{{it}}  + a_{{LF}} \ln L_{{it}} \ln F_{{it}}  + a_{{LO}} \ln L_{{it}} \ln O_{{it}}  + \frac{1}{2}a_{{SS}} \left( {\ln S_{{it}} } \right)^{2}  \\     & \quad  + \,a_{{SF}} \ln S_{{it}} \ln F_{{it}}  + a_{{SO}} \ln S_{{it}} \ln O_{{it}}  + \frac{1}{2}a_{{FF}} \left( {\ln F_{{it}} } \right)^{2}  + a_{{FO}} \ln F_{{it}} \ln O_{{it}}  \\     & \quad  + \,\frac{1}{2}a_{{OO}} \left( {\ln O_{{it}} } \right)^{2}  - \left( {\gamma _{0}  + \gamma _{P} P_{{it}}  + \gamma _{C} C_{{it}}  + \gamma _{{Lb}} Lb_{{it}} } \right)^{2}  + \varepsilon _{{it}}  \\  \end{aligned} $$

where *Y*_*it*_ refers to the vegetable output value per mu of the *i*th city in year *t*. *L*_*it*_, *S*_*it*_, *F*_*it*_ and *O*_*it*_ are growth inputs. They are orderly farmland cost per mu, seed cost per mu, chemical fertilizer quantity per mu and organic fertilizer cost per mu of the *i*th city in year *t*, respectively. The quadratic terms and the interaction terms of four growth inputs are followed. Output value and all of growth inputs are in logarithms.

*P*_*it*_, *C*_*it*_ and *Lb*_*it*_ are the facilitating inputs. They are orderly pesticides, capital and labor of the *i*th city in year *t*, respectively. In Eq. (1), we use different functional forms to reflect the different ways that growth inputs and facilitating inputs contribute to vegetable output. It is worth noting that the magnitude of outputs in Eq. (1) is mainly determined by growth inputs. The effects of facilitating inputs to vegetable output can be written as $\exp \left[ {\left( {\gamma _{0}  + \gamma _{P} P_{{it}}  + \gamma _{C} C_{{it}}  + \gamma _{{Lb}} Lb_{{it}} } \right)^{2} } \right]$, which is defined in the interval [0,1]. This term can measure the impacts of growth conditions on vegetable production such as pests and disasters, and climate changes. Holding the quantity of growth inputs constant, the vegetable output achieves its maximum value if the growth conditions are optimal. Under non-optimal conditions, facilitating inputs are needed and the outputs will be downscaled. Following Guan et al.’s^41^ suggestion, we use the generalized method of moments (GMM) technique to estimate Eq. (1).

Based on the estimated parametric, we can calculate MPVs of chemical fertilizer and pesticide using the following equation:

2 $$ \begin{aligned}   MPV_{{Fit}}  =  & \left( {{\raise0.7ex\hbox{${\partial \ln Y_{{it}} }$} \!\mathord{\left/ {\vphantom {{\partial \ln Y_{{it}} } {\partial \ln F_{{it}} }}}\right.\kern-\nulldelimiterspace} \!\lower0.7ex\hbox{${\partial \ln F_{{it}} }$}}} \right) \times \left( {{\raise0.7ex\hbox{${Y_{{it}} }$} \!\mathord{\left/ {\vphantom {{Y_{{it}} } {F_{{it}} }}}\right.\kern-\nulldelimiterspace} \!\lower0.7ex\hbox{${F_{{it}} }$}}} \right) \times P_{{it}}  \\     =  & \left( {a_{F}  + a_{{LF}} \ln L_{{it}}  + a_{{SF}} \ln S_{{it}}  + a_{{FF}} \ln F_{{it}}  + a_{{FO}} \ln O_{{it}} } \right) \times \left( {{\raise0.7ex\hbox{${Y_{{it}} }$} \!\mathord{\left/ {\vphantom {{Y_{{it}} } {F_{{it}} }}}\right.\kern-\nulldelimiterspace} \!\lower0.7ex\hbox{${F_{{it}} }$}}} \right) \times P_{{it}}  \\  \end{aligned} $$

3 $$ \begin{aligned}   MPV_{{Pit}}  =  & \left( {{\raise0.7ex\hbox{${\partial \ln Y_{{it}} }$} \!\mathord{\left/ {\vphantom {{\partial \ln Y_{{it}} } {\partial P_{{it}} }}}\right.\kern-\nulldelimiterspace} \!\lower0.7ex\hbox{${\partial P_{{it}} }$}}} \right) \times Y_{{it}}  \times P_{{it}}  \\     =  &  - 2 \times \left( {\gamma _{0}  + \gamma _{P} P_{{it}}  + \gamma _{C} C_{{it}}  + \gamma _{{Lb}} Lb_{{it}} } \right) \times \gamma _{P}  \times Y_{{it}}  \times P_{{it}}  \\  \end{aligned} $$

where *MPV*_*Fit*_ and *MPV*_*Pit*_ are MPVs of chemical fertilizer and pesticide, respectively. *P*_*it*_ is the vegetable price per kg of the *i*th city in year *t*. The other symbols have the same meaning as in Eq. (1). We can also calculate MPVs of other inputs.

## Data

Data used in this paper are directly downloaded from the China Agricultural Product Cost–Benefit Compilation (2005–2018) which are issued by the National Development and Reform Commission of China (NDRC). The data covers 34 Chinese cities from mostly all of provinces in China during 2004–2017. This dataset has been widely used by Zhou et al.^32^, Rae et al.^46^, and Jin et al.^47^ to study both Chinese livestock sector and crop sector. A three-stage stratified random sampling procedure is used to get the representative sample counties, villages and finally individual farms. NDRC collected the detailed inputs and output information of vegetable production on farm level, including vegetable output, seed cost, pesticide cost, the quantity and cost of chemical fertilizer (quantity of chemical fertilizer used here is the net quantity calculated after purification) and labor, organic fertilizer cost, land cost and other intermediate input costs per unit mu. To improve the accuracy of the data, bookkeeping method was used and farmers were required to record the inputs which were used several times in each growing duration every time.

**Figure 1:**
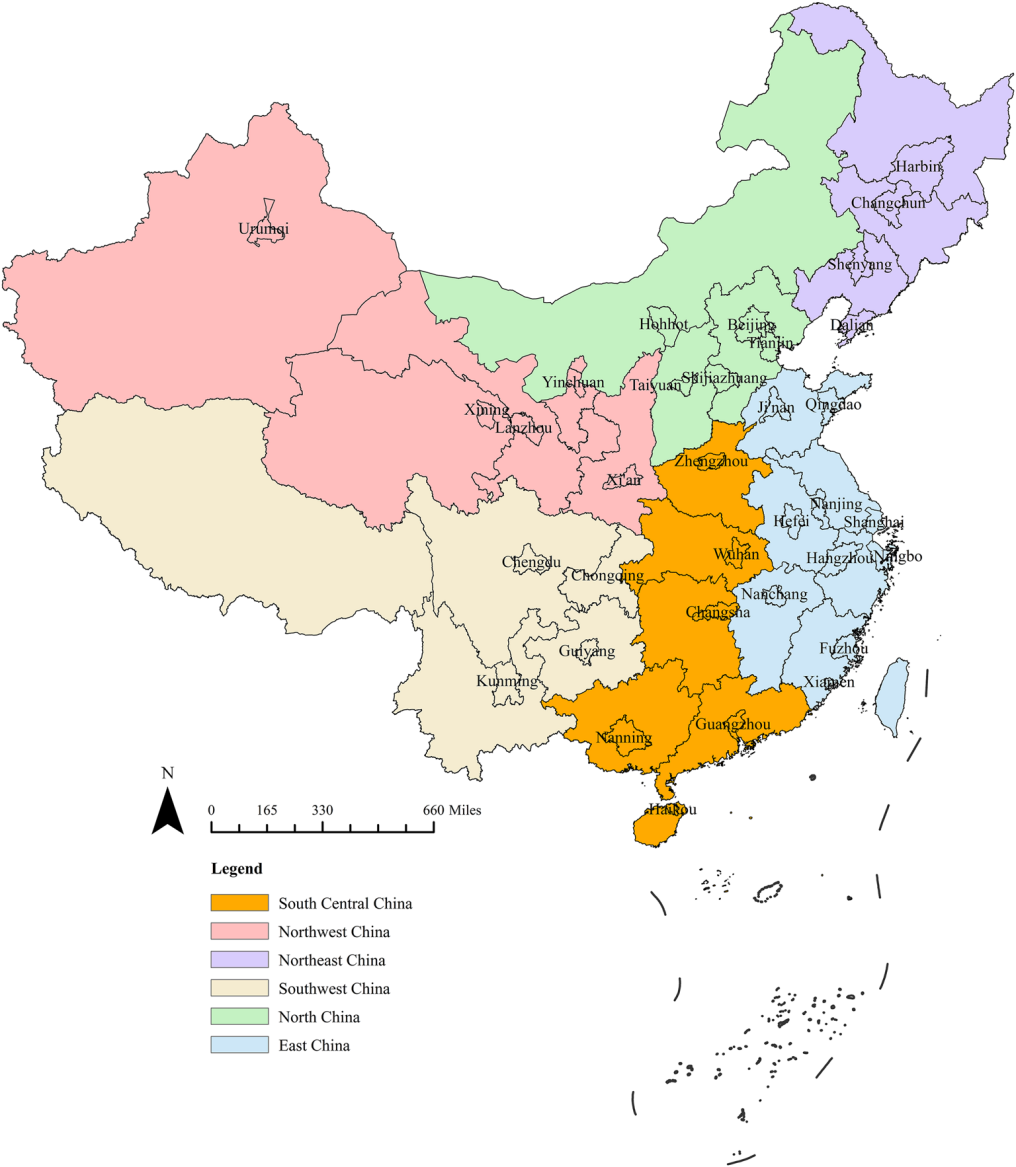
The geographical map of the regions and cities in this study. The map was generated with ArcGIS Desktop version 10.2 (https://desktop.arcgis.com/en). Data of 34 Chinese cities were collected from National Development and Reform Commission of China. All these cities were divided into 6 regions marked with different colors: Beijing, Tianjin, Shijiazhuang, Taiyuan and Hohhot belong to North China (light green); Shenyang, Dalian, Changchun and Harbin belong to Northeast China (purple); Shanghai, Nanjing, Hangzhou, Ningbo, Hefei, Fuzhou, Xiamen, Nanchang, Qingdao and Jinan belong to East China (light blue); Zhengzhou, Wuhan, Changsha, Guangzhou, Nanning and Haikou belong to South Central China (yellow); Chengdu, Guiyang, Kunming and Chongqing belong to Southwest China (light yellow); Xi’an, Lanzhou, Xining, Yinchuan and Urumqi belong to Northwest China (pink).

However, NDRC does not release the farm level data. The data used in this paper are aggregated on city level. We use producer price index of agricultural products in corresponding province to eliminate inflation. There are 34 cities in our sample, divided into 6 regions, including Beijing, Tianjin, Shijiazhuang, Taiyuan and Hohhot in North China, Shenyang, Dalian, Changchun and Harbin in Northeast China, Shanghai, Nanjing, Hangzhou, Ningbo, Hefei, Fuzhou, Xiamen, Nanchang, Qingdao and Jinan in East China, Zhengzhou, Wuhan, Changsha, Guangzhou, Nanning and Haikou in South Central China, Chengdu, Guiyang, Kunming and Chongqing in Southwest China, as well as Xi’an, Lanzhou, Xining, Yinchuan and Urumqi in Northwest China. This paper will focus on cucumbers and tomatoes (including greenhouse and open-field) because these two are dominate vegetable crops in China, accounting for 37% and 33% of China’s total vegetable production, respectively^48^. We show the cities and the regions in a geographical map in Fig. 1.

## Results and discussion

Using large amounts of fertilizers and pesticides on per unit area is one of the dominated factors causing environment pollution in greenhouse production system. However, greenhouse vegetables have higher yield and higher sale price than their counterparts due to the off-season sales. Whether or not greenhouse vegetables have higher overuse degree of agrochemicals compared to open-field vegetables during a growing duration with consideration of both the vegetable’s sale price and yield?

### The status of fertilizer and pesticide application levels

Table 1 presents the status of fertilizer, pesticide and organic fertilizer used by both greenhouse and open-field vegetables, respectively. For chemical fertilizer, farmers use 53.591 kg and 57.452 kg chemical fertilizers on greenhouse tomato and cucumber per mu, which are 4.861 kg and 11.440 kg more than those of open-field tomato and cucumber, respectively. For pesticides, the cost of pesticide per mu for greenhouse cucumber is 35.894 RMB higher than those of open-field cucumber, but no significant differences are observed in pesticide costs for greenhouse and open-field tomatoes. For organic fertilizer, greenhouse tomato and cucumber costs 58.435 RMB and 62.870 RMB more on per mu than those of open-field tomato and cucumber. Overall, chemical fertilizer and pesticide applied on per mu in greenhouse vegetable system are higher than those in open-field system. Based on those data, we may conclude that greenhouse production system would result in more serious environmental problems. However, we also find that greenhouse tomato and cucumber have 652.509 kg and 1278.734 kg higher yield than those of open-field system, respectively. Therefore, it is worth nothing that the differences of agrochemical application between greenhouse and open-field are mostly contrary to those mentioned above, when analyzing the amount of fertilizer and pesticide consumed by per kg of output.

**Table 1:** Application levels of fertilizer and pesticide.

Vegetable		Open-field	Greenhouse	Differences
Tomato	Quantity of chemical fertilizer per mu (kg/mu)	48.729	53.591	− 4.861***
	Pesticide costs per mu (RMB/mu)	149.538	147.425	2.113
	Costs of organic fertilizer per mu (RMB/mu)	148.176	206.612	− 58.435***
	Quantity of chemical fertilizer per kg yield (kg/kg)	0.01124^a^	0.01057	0.00067*
	Pesticide costs per kg (RMB/kg)	0.036	0.029	0.006***
	Costs of organic fertilizer per kg (RMB/kg)	0.034	0.039	− 0.005***
	Yield per mu (kg/mu)	4517.629	5170.139	− 652.509***
	Output value per mu (RMB/mu)	4864.846	7036.054	− 2171.208***
	Price per kg (RMB/kg)	0.795	0.962	− 0.167***
Cucumber	Quantity of chemical fertilizer per mu (kg/mu)	46.012	57.452	− 11.440***
	Pesticide costs per mu (RMB/mu)	119.180	155.073	− 35.894***
	Costs of organic fertilizer per mu (RMB/mu)	133.488	196.358	− 62.870***
	Quantity of chemical fertilizer per kg yield (kg/kg)	0.01224^a^	0.01151	0.00073*
	Pesticide costs per kg (RMB/kg)	0.033	0.030	0.003**
	Costs of organic fertilizer per kg (RMB/kg)	0.035	0.038	− 0.003**
	Yield per mu (kg/mu)	4018.586	5297.321	− 1278.734***
	Output value per mu (RMB/mu)	3933.373	6933.083	− 2999.710***
	Price per kg (RMB/kg)	0.734	0.935	− 0.201***

*,**,*** indicate statistically significance at the 10%, 5% and 1%, respectively. 1 hectare = 15 mu. Quantity of chemical fertilizer used here is the net quantity calculated after purification.

^a^We keep 5 decimal places due to the small value.

**Figure 2:**
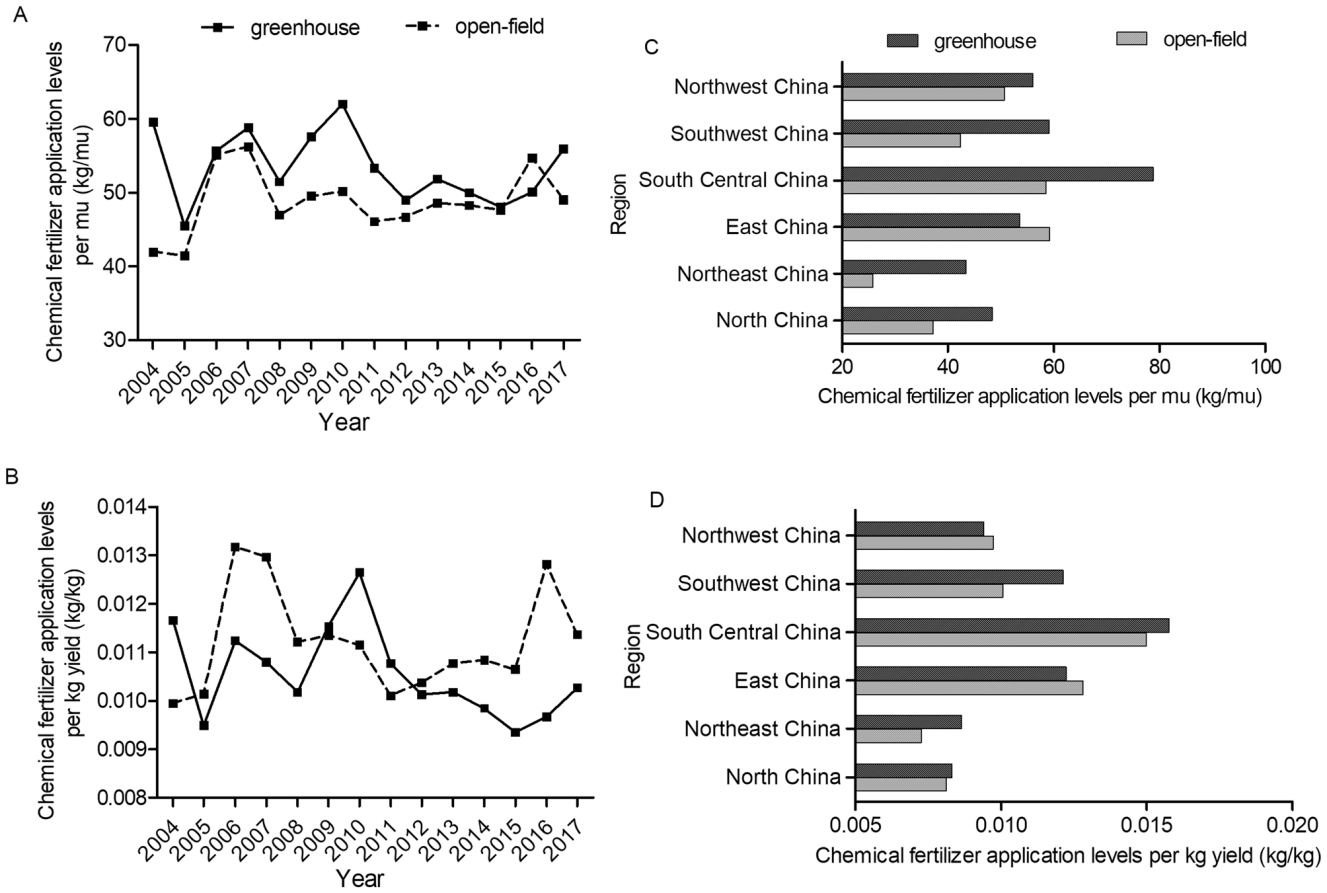
Chemical fertilizer application levels on greenhouse and open-field tomatoes. (**A**) and (**B**) presented chemical fertilizer application levels per mu and per kg yield during 2004–2017, respectively. (**C**) and (**D**) presented the average values of chemical fertilizer application levels (per mu and per kg yield, respectively) across six regions in 2017.

**Figure 3:**
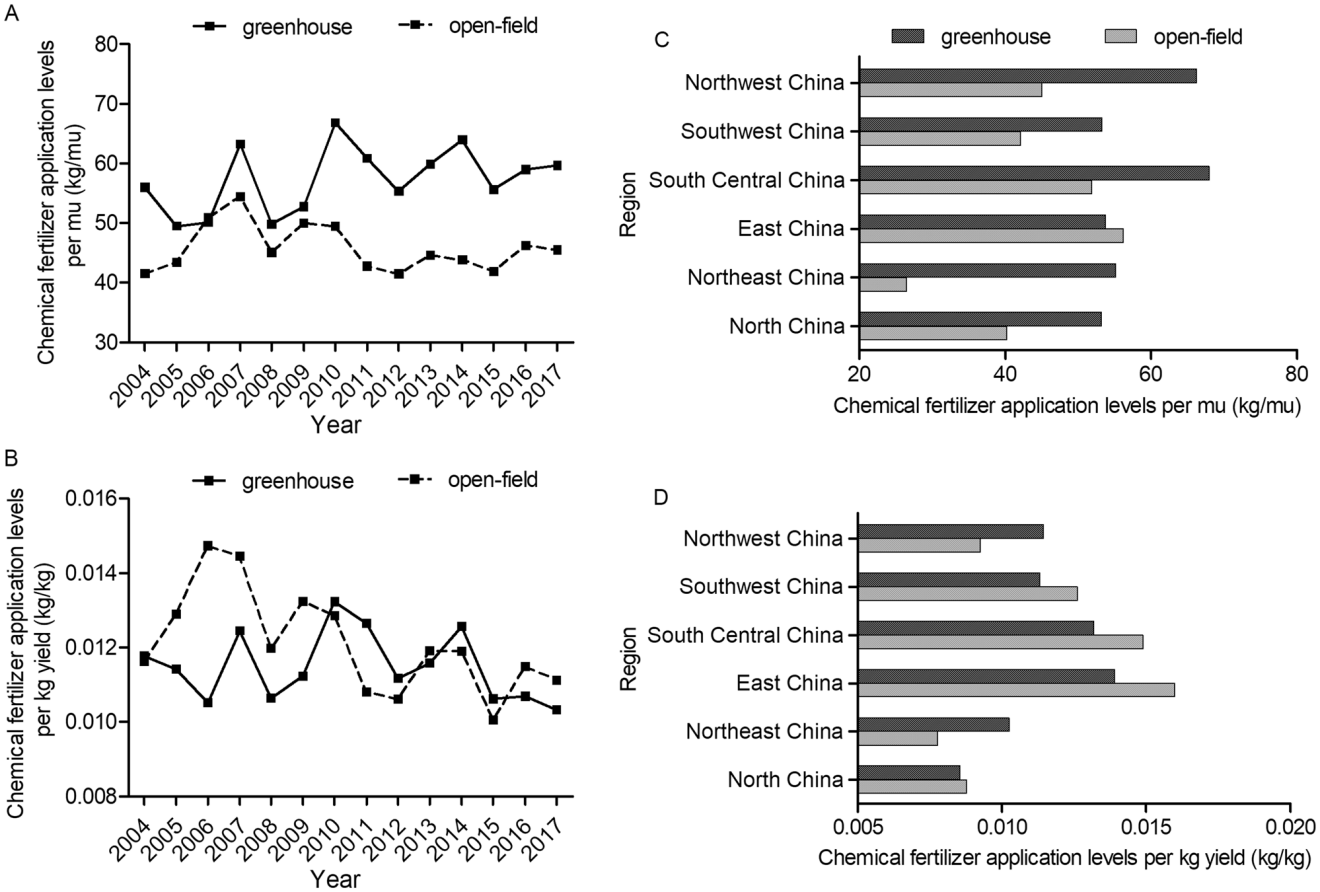
Chemical fertilizer application levels on greenhouse and open-field cucumbers. (**A**) and (**B**) presented chemical fertilizer application levels per mu and per kg yield during 2004–2017, respectively. (**C**) and (**D**) presented the average values of chemical fertilizer application levels (per mu and per kg yield, respectively) across six regions in 2017.

**Figure 4:**
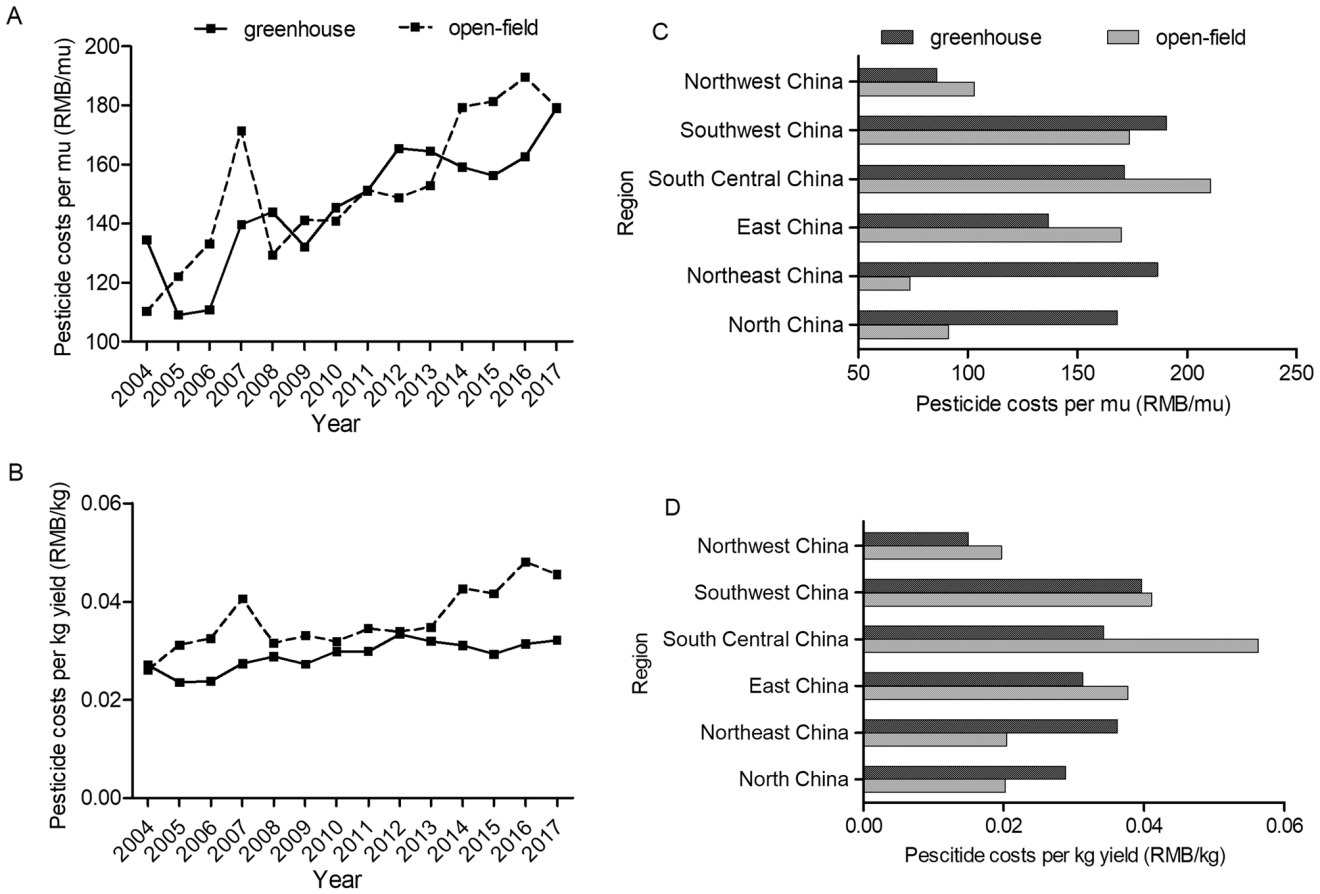
Costs of organic fertilizers on greenhouse and open-field tomatoes. (**A**) and (**B**) presented costs of organic fertilizers per mu and per kg yield during 2004–2017, respectively. (**C**) and (**D**) presented the average values of organic fertilizers costs (per mu and per kg yield, respectively) across six regions in 2017.

**Figure 5:**
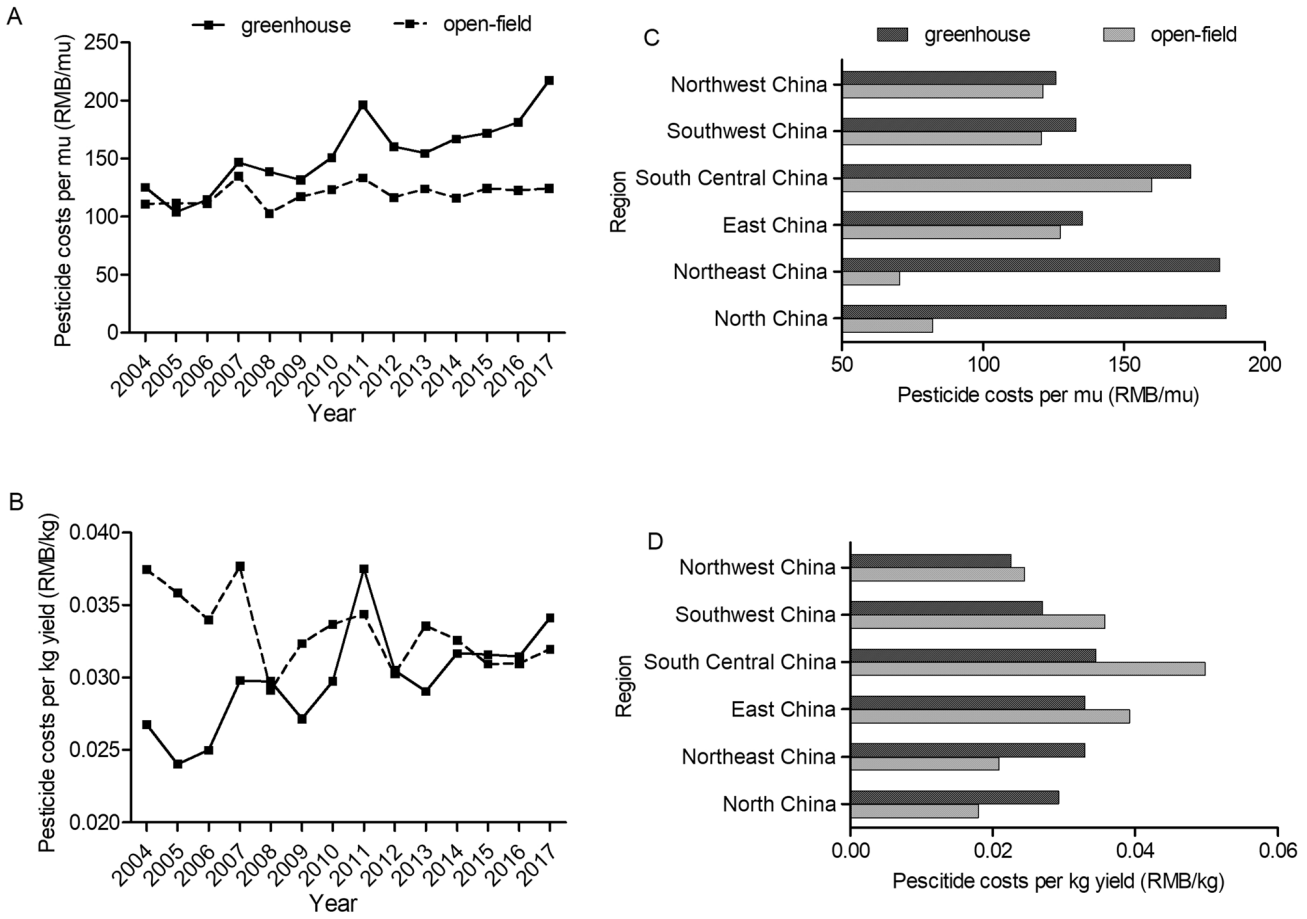
Costs of organic fertilizers on greenhouse and open-field cucumbers. (**A**) and (**B**) presented costs of organic fertilizers per mu and per kg yield during 2004–2017, respectively. (**C**) and (**D**) presented the average values of organic fertilizers costs (per mu and per kg yield, respectively) across six regions in 2017.

Detailed comparison of the fertilizer application levels between greenhouse and open-field is presented in Figs. 2, 3, 4 and 5. For both tomato and cucumber, regardless of greenhouse or open-field, chemical fertilizer application levels have experienced fluctuations during 2004–2017 without apparent increase or decrease trends. More specifically, for most years during 2004–2017, the fertilizer consumption per mu by greenhouse vegetable is generally higher (Figs. 2A and 3A), but the fertilizer consumption per kg output by greenhouse vegetable is mostly lower compared to open-field vegetable (Figs. 2B and 3B). These results are consistent with the statistical analysis results in Table 1, indicating that greenhouse production system has higher input to output ratio of chemical fertilizer compared to that of open-field production system. However, different from chemical fertilizer, the costs of organic fertilizer per mu and per kg yield in greenhouse are both significantly higher than that in open-field (Figs. 4 and 5) during 2004–2017 with a slightly increase trend.

A large number of studies have proved that about 40–60% of crop yield is attributable to chemical fertilizer^49,50^, and organic fertilizer is mainly used to maintain soil fertility and improve soil structure^51^. Yan and Gong^52^ documented that the nutrient use efficiency of chemical fertilizer was higher than that of organic fertilizer which cannot improve crop yield in the current season but would benefit crop yield in the long term. Our study finds that both chemical and organic fertilizers applied in greenhouse vegetable system are higher than in open-field system, which may contribute to the higher yield in greenhouse. Although Ti et al.^53^ had stated that fertilizer (nitrogen) use efficiency in greenhouse system is lower than in open-field system, this study proves that greenhouse vegetable system could consume less chemical fertilizer than open-field system to harvest the same yield in reality. One possible explanation is that greenhouse system could provide regulated and controlled growing conditions for vegetables. Therefore, compared to open-field vegetables, greenhouse vegetables could have longer harvest duration, produce more outputs and higher input to output ratio.

Figures 2, 3, 4 and 5 also shows the chemical fertilizer and organic fertilizer consumption across six regions in China. This study finds that higher amounts of chemical fertilizer are applied per mu in Central China for both greenhouse and open-field vegetable systems, while Northeast China use the lowest amounts of chemical fertilizer for both systems (Figs. 2C and 3C) due to the fertile black soil. Meanwhile, the highest inputs (per mu) of organic fertilizer are observed in Northwest China, and the lowest inputs are in Southwest China for the two vegetable systems (Figs. 4C and 5C). Previous studies analyzed the regional disparity in fertilizer input of main crops in China and draw similar conclusion: the input intensity showed a decreasing trend from east to west^54,55^.

**Figure 6:**
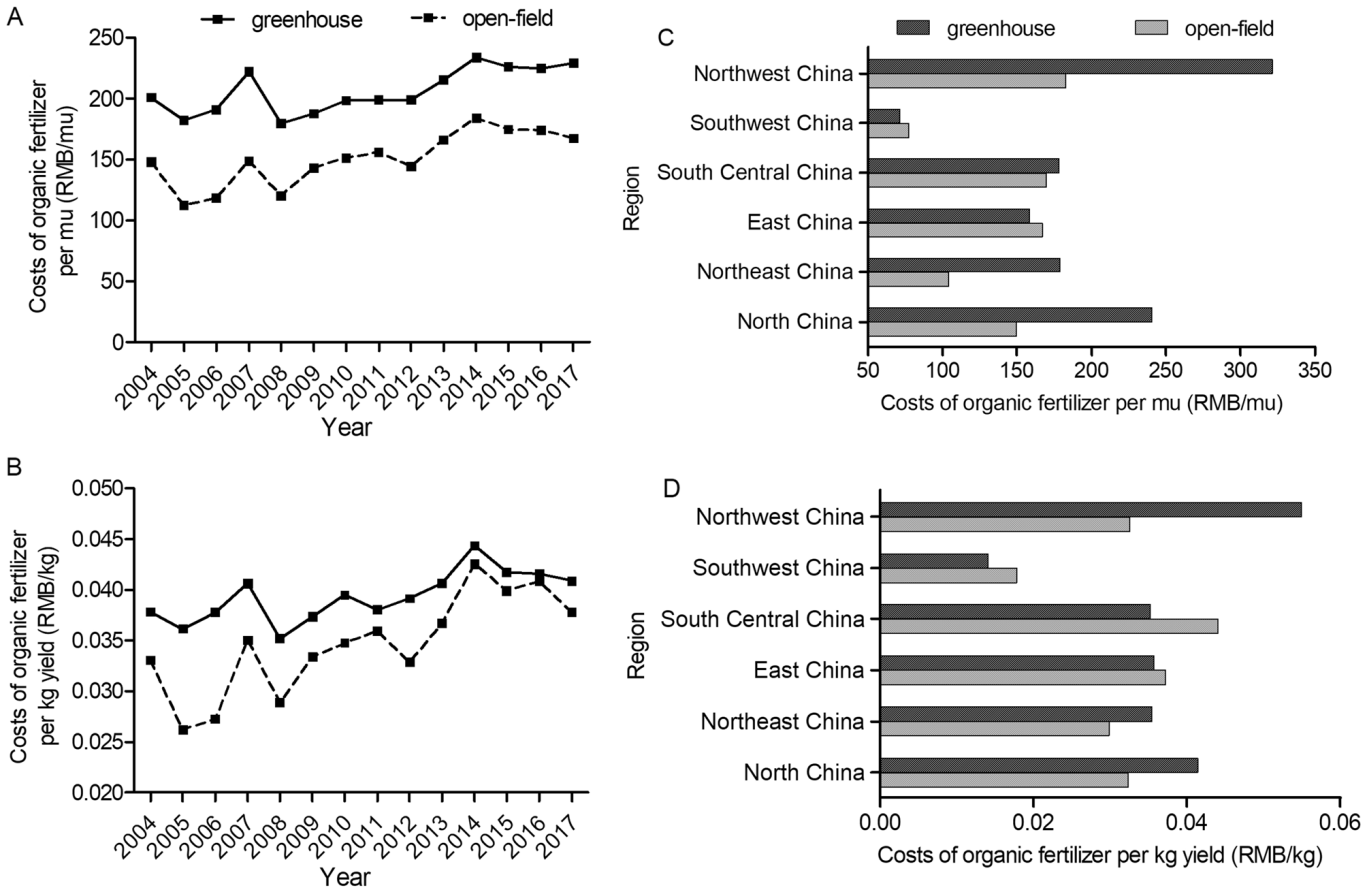
Pesticide costs for greenhouse and open-field tomatoes. (**A**) and (**B**) presented pesticide costs per mu and per kg yield during 2004–2017, respectively. (**C**) and (**D**) presented the average values of pesticide costs (per mu and per kg yield, respectively) across six regions in 2017.

**Figure 7:**
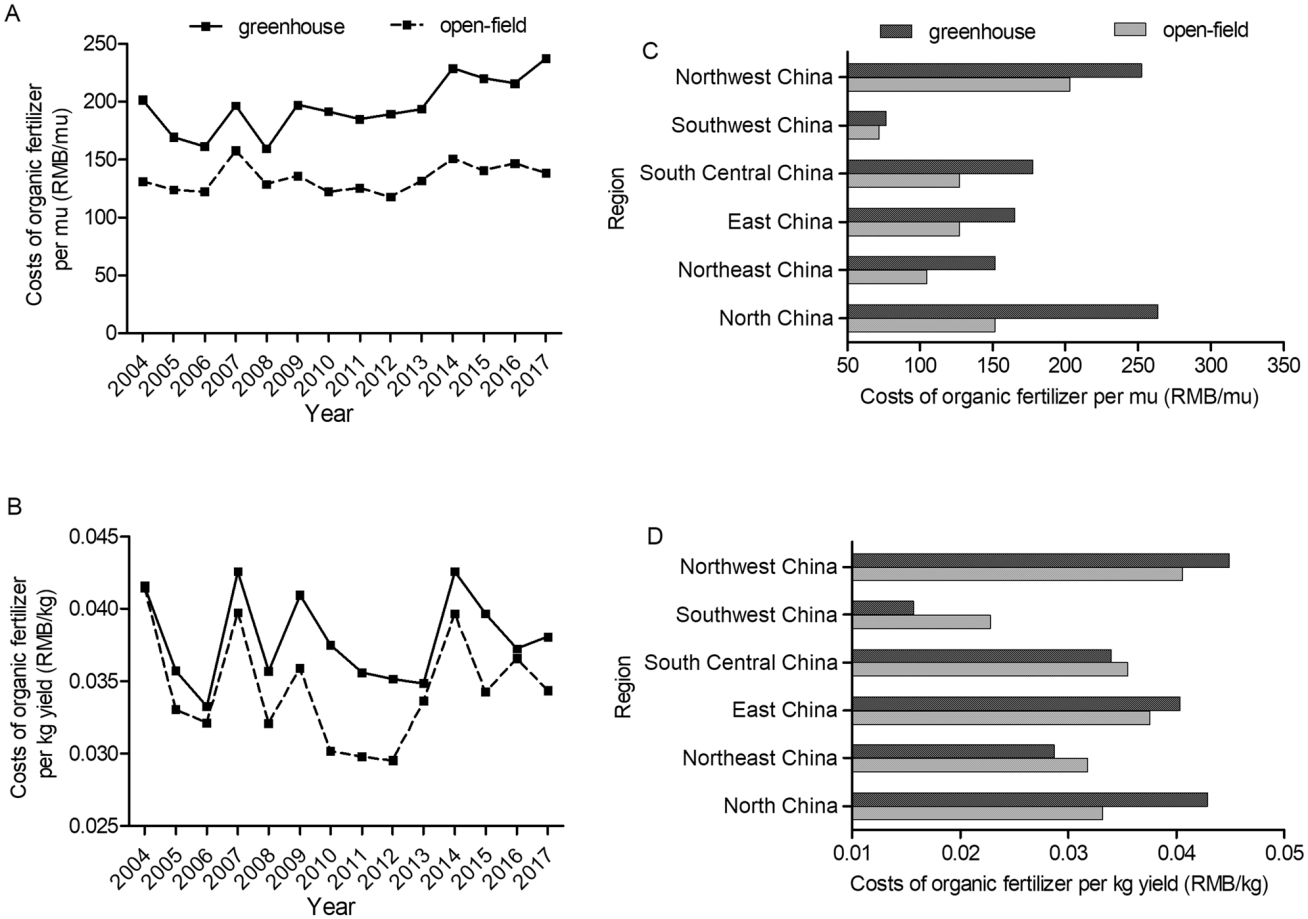
Pesticide costs for greenhouse and open-field cucumbers. (**A**) and (**B**) presented pesticide costs per mu and per kg yield during 2004–2017, respectively. (**C**) and (**D**) presented the average values of pesticide costs (per mu and per kg yield, respectively) across six regions in 2017.

In addition, Figs. 6 and 7 depict pesticide costs for tomato and cucumber in different regions and over years. Greenhouse production system is prone to invasions of pests and pathogens due to the characteristics of high humidity, high temperature and continuous cropping in greenhouse environment^25^. It’s inevitable to apply lots of pesticides. The results show that pesticide costs per mu for both greenhouse tomato and cucumber have experienced long-term increase trend with considerable fluctuations during 2004–2017. Pesticide costs per mu for open-field tomato also show an overall increase trend with fluctuation during 2004–2017, while pesticide costs per mu for open-field cucumber maintain about 120 RMB per mu. Due to the higher yield in greenhouse, pesticide costs for per kg greenhouse tomato and cucumber are mostly lower than those for open-field tomato during 2004–2017 which is consistent with the results in Table 1. Significant differences also exist in pesticide costs across regions. More specifically, Central China consumes relative high levels of pesticide for both greenhouse and open-field vegetable systems.

To summary, farmers use more chemical fertilizers and pesticides on per mu whereas use less on per kg greenhouse vegetables compared to open-field vegetables. We may conclude that the input to output ratio of agrochemicals for greenhouse vegetables is not lower than that of open-field vegetables during one growth duration with yield into consideration. However, why do the pollution caused by agrochemicals in greenhouse system always arise people’s concerns? There are two possible explanations: First, greenhouse system could be used to produce vegetables all year round without rest for soil due to the regulated and controlled conditions. Second, although the input to output ratio of agrochemicals is high for greenhouse system, the pollution caused by agrochemicals may be more serious due to the special microclimate environment of high temperature and humidity. In addition, the sale price of greenhouse vegetables during off season is significantly higher. Therefore, farmers have economics incentives to produce more vegetables by using more agrochemicals, which may result in pollution.

### Allocative efficiency of fertilizers and pesticides

In this part, we evaluate the allocative efficiency of agrochemicals for both greenhouse vegetables and open-field vegetables by calculating the MPVs of fertilizers and pesticides and make comparison with their corresponding prices with sale price into consideration.

Following Guan et al.’s^41^ work, we specify an asymmetric translog production function by integrating both agronomic principles and agricultural economics concepts. Table 2 reports the estimators of the asymmetric translog production function. We use GMM technique and take the inputs of last year as instrumental variable to avoid endogenous problems when we estimate the production function. Among the growth inputs, only the quadratic term of seed contributes to the growth of cucumber outputs. This is because selecting the input variables is a trade-off between applying more inputs to get more useful information and increasing the risk of multicollinearity^30,32^. However, the linear terms of land, seed, organic fertilizer, and some of the interaction terms have statistically significant impacts on tomato outputs. Among the facilitating inputs, both pesticide and labor have significant impacts on cucumber outputs, and both capital and labor have impacts on tomato outputs. The positive coefficients for facilitating inputs indicate that farmers are likely to harvest less output if they apply more facilitating inputs in production process under non-optimal conditions.

**Table 2:** Estimation results of the asymmetric translog production function.

Inputs	Tomato	Cucumber
land	− 3.436*** (0.95)	− 0.629 (0.65)
seed	1.669** (0.84)	− 0.501 (0.40)
fertilizer	− 0.277 (0.63)	− 0.497 (0.62)
Organic fertilizer	1.303** (0.66)	0.694 (0.58)
Land*land	0.279 (0.19)	0.096 (0.09)
Land*seed	− 0.011 (0.13)	0.004 (0.11)
Land*fertilizer	0.466*** (0.16)	0.137 (0.14)
Land*organic fertilizer	0.078 (0.10)	− 0.087 (0.10)
Seed*seed	− 0.091 (0.09)	0.142* (0.07)
Seed*fertilizer	− 0.069 (0.09)	0.077 (0.07)
Seed* organic fertilizer	− 0.216* (0.12)	− 0.076 (0.08)
Fertilizer*fertilizer	− 0.064 (0.20)	− 0.032 (0.09)
Fertilizer* organic fertilizer	− 0.266** (0.11)	− 0.065 (0.09)
ORGANIC fertilizer * organic fertilizer	0.063 (0.07)	0.096 (0.08)
Pesticides	0.005 (0.01)	− 0.016* (0.01)
Capital	0.061*** (0.01)	0.013 (0.01)
Labor	0.032** (0.01)	0.036** (0.02)
Open-field relative to greenhouse	− 0.165* (0.09)	0.247*** (0.07)
Time trends	0.042*** (0.01)	0.036*** (0.01)
Constants	2.285*** (0.42)	2.480*** (0.35)
Number of observations	577	583

Growth inputs include land, seed, chemical fertilizer and organic fertilizer. Facilitating inputs include pesticide, capital and labor. Standard errors are shown in parentheses.

*,**,*** indicate statistical significance at the 10%, 5% and 1%, respectively.

Hopper^29^ firstly compared the MPVs of agricultural inputs with their prices to support the hypothesis that Indian farmers are “poor but efficient”: Indian farmers allocate their input efficiently because the additional output value produced by using one more unit of input equals to the input’s price. Based on the estimated parametric in Table 2, MPVs of fertilizers and pesticides are calculated by using Eqs. (2) and (3), and the results are reported in Table 3. The null hypothesis that MPVs of chemical fertilizer equal to zero is not rejected at 10% significance level indicating overuse of chemical fertilizer for both greenhouse and open-field tomatoes. However, we find that one kg chemical fertilizer applied on greenhouse cucumber could produce output value of 4.052 RMB which is statistically significant different from zero and insignificant from its price. This implies that farmers allocate chemical fertilizer efficiently on greenhouse cucumber. However, farmers overuse chemical fertilizer on open-field cucumber.

**Table 3:** Marginal product values and the prices of fertilizer and pesticide.

Vegetable inputs	Tomato		Cucumber	
	Open-field	Greenhouse	Open-field	Greenhouse
**Chemical fertilizer**				
Marginal production value (MPV:RMB)	0^a^	1.711	0^a^	4.052
Input price (IP: RMB)	4.769	5.267	4.745	5.833
*P* value (H0: MPV = 0)	NA	0.503	0.003	0.072
*P* value (H0: MPV = IP)	0.000	0.080	0.000	0.216
**Organic fertilizer**				
Marginal production value (MPV:RMB)	14.576	9.880	23.059	20.173
Input price (IP: RMB)	1.398	1.420	1.399	1.419
*P* value (H0: MPV = 0)	0.000	0.000	0.000	0.000
*P* value (H0: MPV = IP)	0.000	0.000	0.000	0.000
**Pesticide**				
Marginal production value (MPV:RMB)	0^a^	0^a^	0^a^	0^a^
Input price (Pesticide price index)	1.173	1.177	1.172	1.176
*P* value (H0: MPV = 0)	NA	NA	NA	NA
*P* value (H0: MPV = IP)	0.000	0.000	0.000	0.00

One more unit of agrochemical does not produce any vegetables. NA refers to no value.

^a^The input does not significantly affects the output, therefore, the MPV is zero.

Based on the results reported in Table 2, pesticide has no significant effects on tomato output, thus, the MPV of pesticide used in tomato production regardless of greenhouse or open-field is zero. This implies that pesticide is overused for both greenhouse and open-field tomatoes. Similarly, one RMB increase in pesticide costs will negatively affect the output values implying overuse for both open-field and greenhouse cucumbers. It is consistent with the damage-abating role of pesticide. In other words, the more pesticides used the lower the yield due to the severe pests and diseases. The difference in pesticide costs between tomato and cucumber is expected. The special smell of tomato plants makes it more resistant to diseases and insect pests than cucumber plants.

The analysis of organic fertilizer shows that one RMB increase in organic fertilizer will produce output value of more than 10 RMB for both greenhouse and open-field tomato or cucumber, indicating underuse of organic fertilizers. Therefore, increasing the application of organic fertilizer will contribute greatly to the vegetable output. However, it is worth emphasizing that fertilizer with manure and especially animal feces might result in high concentrations of heavy metals and antibiotic in soils^8,18^. Higher inputs of organic fertilizer in greenhouse lead to greater potential threat to environment and human health.

To summary, MPVs of chemical fertilizer and pesticide are significantly lower than their prices for both greenhouse vegetables and open-field vegetables excluding those of chemical fertilizer applied on greenhouse cucumber. These findings reflect that chemical fertilizer and pesticide are generally overused in vegetable sector with sale price of vegetables into consideration. However, the higher MPVs of chemical fertilizer for greenhouse vegetables imply that greenhouse vegetables do not have higher overuse degrees of chemical fertilizer than open-field vegetables. The results in this part furtherly document that greenhouse vegetables do not have lower allocation efficiency for chemical fertilizers than open-field vegetables with vegetables’ sale price and yield into consideration. Therefore, the serious pollution caused by agrochemicals in greenhouse vegetables may attribute to the year-round production model because farmers usually take greenhouse production system as an important method to increase outputs by growing vegetables several times in greenhouse per year. The overuse degree and environment pollutions could be significantly alleviated in greenhouse production system if farmers just take advantage of the function to provide controlled growing conditions during off-season rather than year-round production.

One possible policy implication is that reducing the number of production rotations may be an effective method to alleviate the overuse of agrochemicals used in greenhouse vegetables. To produce more and more vegetables, farmers not only use the greenhouse to provide the regulated and controlled growing conditions in off-season but also produce vegetables in the other seasons. We found that more and more farmers use greenhouse to grow only one season of tomato per year in off-season to protect the soil quality in the south of Jiangsu province. In addition, to balance environment protection and vegetable production, modern farming and management practices should be introduced to vegetable industry, such as biological control, rational and precise fertilization, which are much easier to implement in greenhouse. We also found that farmers replaced chemical fertilizer and pesticide with organic fertilizer and biological control methods to protect the soil and improve the quality of products in the south of Jiangsu province.

## Data Availability

Data is available for revision.

## Abbreviations

GMM: Generalized method of moments
MPVs: Marginal product values
NDRC: National Development and Reform Commission of China
